# Holographic patient tracking after bed movement for augmented reality neuronavigation using a head-mounted display

**DOI:** 10.1007/s00701-021-04707-4

**Published:** 2021-01-29

**Authors:** T. Fick, J.A.M. van Doormaal, E.W. Hoving, L. Regli, T.P.C. van Doormaal

**Affiliations:** 1 Department of Neuro-oncology, Princess Máxima Center for Pediatric Oncology, Heidelberglaan 25, 3584 CS Utrecht, The Netherlands; 2 Department of Oral and Maxillofacial surgery, University Medical Centre Utrecht, Heidelberglaan 100, 3584 CX Utrecht, The Netherlands; 3 Department of Neurosurgery, University Medical Centre Utrecht, Heidelberglaan 100, 3584 CX Utrecht, The Netherlands; 4 Department of Neurosurgery, Clinical Neuroscience Center, University Hospital Zurich, University of Zurich, Rämistrasse 100, 8091 Zürich, Switzerland

**Keywords:** Patient tracking, Augmented reality, Head-mounted display

## Abstract

**Background:**

Holographic neuronavigation has several potential advantages compared to conventional neuronavigation systems. We present the first report of a holographic neuronavigation system with patient-to-image registration and patient tracking with a reference array using an augmented reality head-mounted display (AR-HMD).

**Methods:**

Three patients undergoing an intracranial neurosurgical procedure were included in this pilot study. The relevant anatomy was first segmented in 3D and then uploaded as holographic scene in our custom neuronavigation software. Registration was performed using point-based matching using anatomical landmarks. We measured the fiducial registration error (FRE) as the outcome measure for registration accuracy. A custom-made reference array with QR codes was integrated in the neurosurgical setup and used for patient tracking after bed movement.

**Results:**

Six registrations were performed with a mean FRE of 8.5 mm. Patient tracking was achieved with no visual difference between the registration before and after movement.

**Conclusions:**

This first report shows a proof of principle of intraoperative patient tracking using a standalone holographic neuronavigation system. The navigation accuracy should be further optimized to be clinically applicable. However, it is likely that this technology will be incorporated in future neurosurgical workflows because the system improves spatial anatomical understanding for the surgeon.

**Supplementary Information:**

The online version contains supplementary material available at 10.1007/s00701-021-04707-4.

## Introduction

Infrared (IR) navigation systems are broadly used in neurosurgery. These systems guide the surgeon by providing anatomical information about the surgical field and surroundings and allow for a minimal and direct approach to the surgical target [2, 10].

Neuronavigation systems work on two main principles: patient-to-image registration and patient tracking after bed movement. For patient-to-image registration, a transformation matrix has to be calculated between image-space and physical space in order for them to overlap. This is usually performed through surface matching or point-based matching, where fiducials in physical-space and image-space are matched using an iterative closest point algorithm. Patient tracking is the alteration of image space after patient movement in physical space, with the purpose of correcting registration.

Augmented reality is a technology that superimposes a virtual image into the user’s view of the real world. AR-HMDs have seen a major development over the recent years and interest has been shown especially in neurosurgery [3, 12]. As neuronavigation systems, AR-HMDs have two advantages compared to conventional neuronavigation (CN): First, anatomy is shown in a stereoscopic 3D manner so the surgeon does not need to translate the anatomy from a 2-D external screen to the 3-D surgical field, theoretically reducing the chance of interpretation error and leaving more room mentally to concentrate on other surgical tasks. Second, images can be superimposed directly onto the surgical work field, diminishing attention shifts which makes surgical tasks more efficient [4]. To use an AR-HMD as a neuronavigation system, two main principles need to be developed: patient-to-image registration and patient tracking after bed movement. The first has been extensively investigated and several registration methods have been suggested [5–7]. The latter has, to the best of our knowledge, not yet been investigated within the neurosurgical field.

We designed a reference array for use with an AR-HMD to reposition the holograms after registration according to the movements of the head in the Mayfield. Furthermore, we improved our previously published point-based registration method by optimizing the pointer for better accuracy [13]. We present a proof-of-concept on three patients undergoing a neurosurgical procedure to illustrate the complete workflow of a neuronavigation system using an AR-HMD.

## Methods

### Inclusion

Patients were included if they were admitted to the study center for an intracranial neurosurgical procedure with the use of standard IR neuronavigation and scheduled within the period of 24–28 February 2020. All data was collected from our institutional ongoing prospective patient registry as approved by our local ethics committee (KEK 2017–01120). All patients were informed on the purpose and the course of action for the study preoperatively, and all provided informed consent prior to inclusion. No diagnostic or treatment decisions were made based on the holographic navigation system, and registration and measurements were performed simultaneously with the setup of standard neuronavigation. Standard neuronavigation was used for the remaining procedure.

### Segmentation

**Fig. 1:**
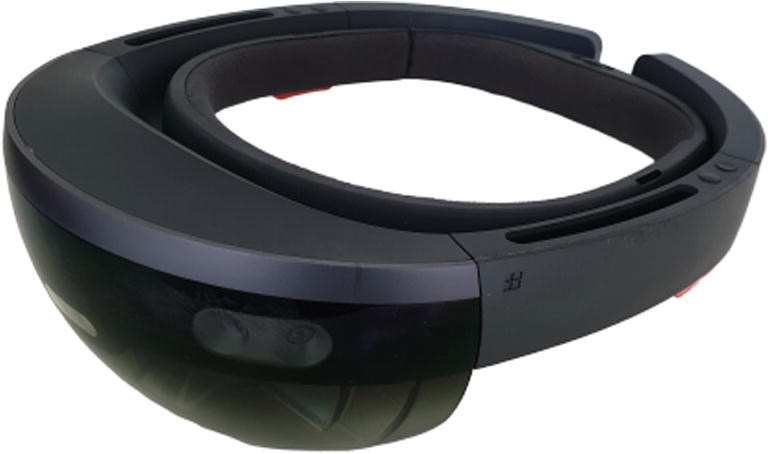
Augmented reality head-mounted display (HoloLens, Microsoft, Redmond, USA)

For each case, the relevant anatomical structures were segmented from CT and MRI scans using medical segmentation software (3D Slicer, Massachusetts Institute of Technology, Boston, USA). For skin and bones, we used a threshold-based segmentation and postprocessed the skin with a hollowing function to reduce the file size of the holograms. The brain and ventricles were segmented with fast marching which after adding some reference points using a paintbrush, expands the selected segment to regions that have similar intensity. Vessels were segmented using the tubing function. The tumors were segmented using a threshold-based segmentation in a predefined region of interest. Anatomical landmarks were defined as registration points. All segmentations and landmarks were exported as .stl files and combined in a single holographic scene. This scene was uploaded to our custom neuronavigation software on a wearable AR-HMD (Hololens 1, Microsoft, Redmond, USA) (Fig. 1).

### Software

We programmed our dedicated holographic navigation software in a real-time 3D development platform (Unity, Unity Technologies, San Francisco, USA) using C++ and C# and an integrated development environment (Visual Studio, Microsoft, Redmond, USA). We used an image recognition library (Vuforia, PTC, Boston, USA) to integrate marker tracking. Using the software, the surgeon can summon, manipulate, and register patient holograms that are directly superimposed over the real field-of-view. The software and AR-HMD work completely independent without the use of external devices and is not connected to the existing CN system in any way.

### Registration

**Fig. 2:**
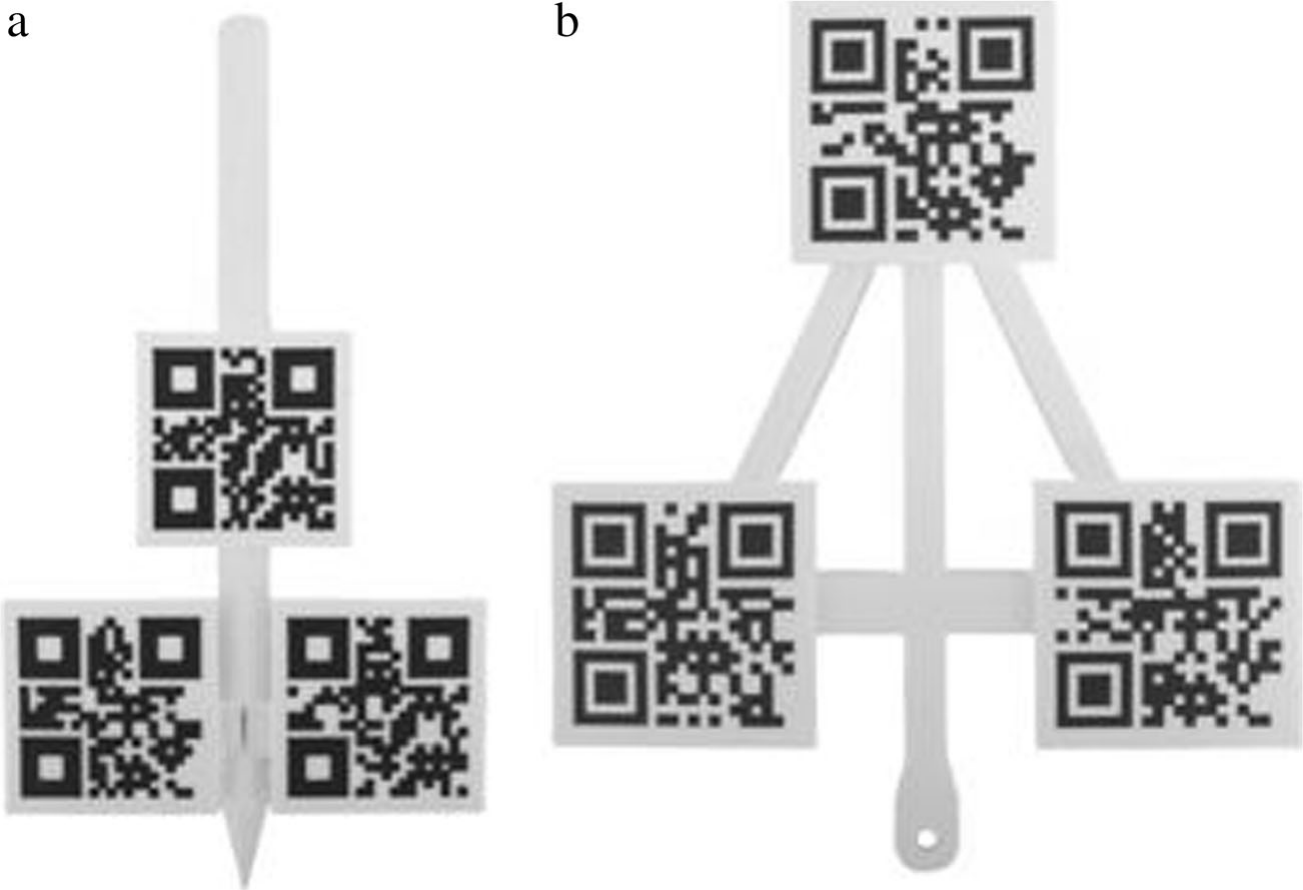
(a) Custom probe for point-based registration (b) Reference array for patient tracking

Registration was conducted using rigid point-based matching with anatomical landmarks. For this, we designed a custom probe (Fig. 2a) that was tracked and overlaid by a virtual pointer by the AR-HMD using X-, Y-, and Z-position detection and point-based matching of the three visual markers. This probe was fabricated using a 3D printer (Ultimaker s5, Ultimaker, Geldermalsen, The Netherlands) in polylactic acid. Using this probe, the patient was then consequently tipped on all defined registration landmarks. A digital point was added to each landmark using the voice command “point.” After pressing the button “match,” the hologram was matched on the real patient using an iterative closest point algorithm. The AR-HMD then calculated the accuracy of the match using the fiducial registration error (FRE), which is defined as the root-mean-square distance between recognized fiducial positions and their homologous virtual fiducial positions after registration.

### Reference array

**Fig. 3:**
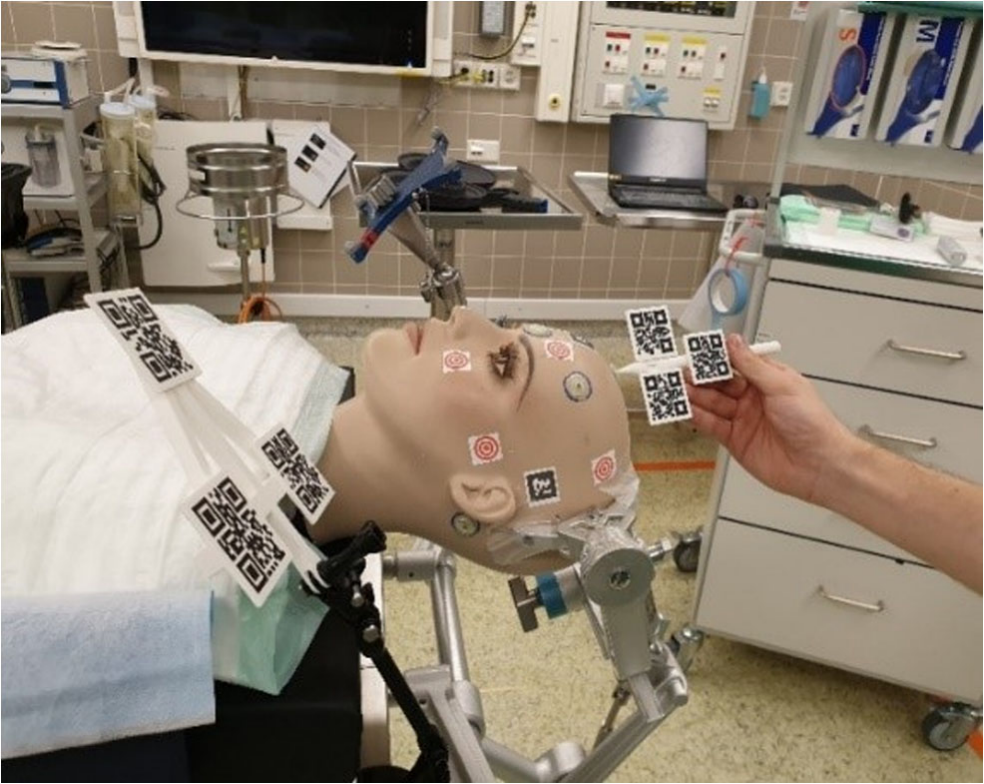
Overview of complete setup on a phantom including custom probe and reference array

We designed a reference array (Fig. 2b) that could be used to reposition the hologram after patient movement. This reference array accommodated three visual markers in a triangular formation, and was fabricated in polylactic acid using a 3-D printer (Ultimaker s5, Ultimaker, Geldermalsen, The Netherlands). The array was directly fixed on the head clamp using a pinching mechanism and had several pivot points to enable the surgeon to adjust the array’s position. When looking at the reference array with the AR-HMD, the location of the reference array was calculated automatically using X-, Y-, and Z-position detection and point-based matching of the 3 markers. After initial registration, the surgeon could “lock” the hologram to the reference array by looking at the array markers until they are recognized by the AR-HMD. After bed movement, the surgeon could correct the hologram position by looking at the array markers again, which moves the complete holographic scene to the new position. An overview of the complete setup is shown with a phantom in Fig. 3. Additional digital content is provided which demonstrates the complete workflow in all three patients (Online Resource 1).

## Results

### Patient 1

**Fig. 4:**
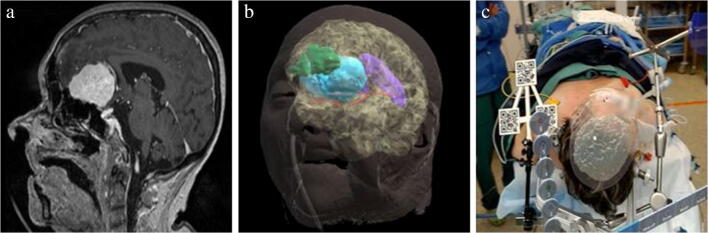
(a) MRI-image of olfactory meningioma, sagittal view (b) Segmentation (c) Holograms after registration as seen through the HoloLens

Patient 1 was an 80-year old female who underwent an olfactory meningioma resection. Based on MRI-imaging (Fig. 4a), holograms were made of the skin, brain, meningioma, ventricles, frontal sinus, and anterior artery complex (Fig. 4b). Nine anatomical landmarks were chosen on the hologram of the skin for registration: medial and lateral canthus of both eyes, nasal bridge, proximal part of the philtrum, a distinctive part of the antihelix on both sides, and the inion. Two registrations were performed with an FRE of 10.0 mm and 9.0 mm (Fig. 4c). After the second registration, the bed was moved upwards. The reference array was tracked again and the hologram repositioned with no visual change in accuracy of registration in the X-, Y-, and Z-direction.

### Patient 2

**Fig. 5:**
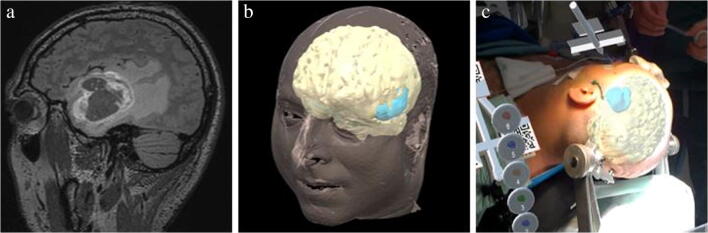
(a) MRI-image of GBM, sagittal view (b) Segmentation (c) Holograms after registration as seen through the HoloLens

Patient 2 was a 48-year-old male who underwent a left temporal glioblastoma multiforme (GBM) resection. Based on MRI-imaging (Fig. 5a), holograms were made of the skin, brain and GBM (Fig. 5b). Eight anatomical landmarks were chosen: medial and lateral canthus of both eyes, nasal bridge, proximal part of the philtrum, and a distinctive part of the antihelix on both sides. Two registrations were performed with an FRE of 8.4 mm and 9.9 mm (Fig. 5c). After each registration, the bed was moved upwards, and the reference array was tracked. Both times, the hologram repositioned with visually no change in accuracy of registration.

### Patient 3

**Fig. 6:**
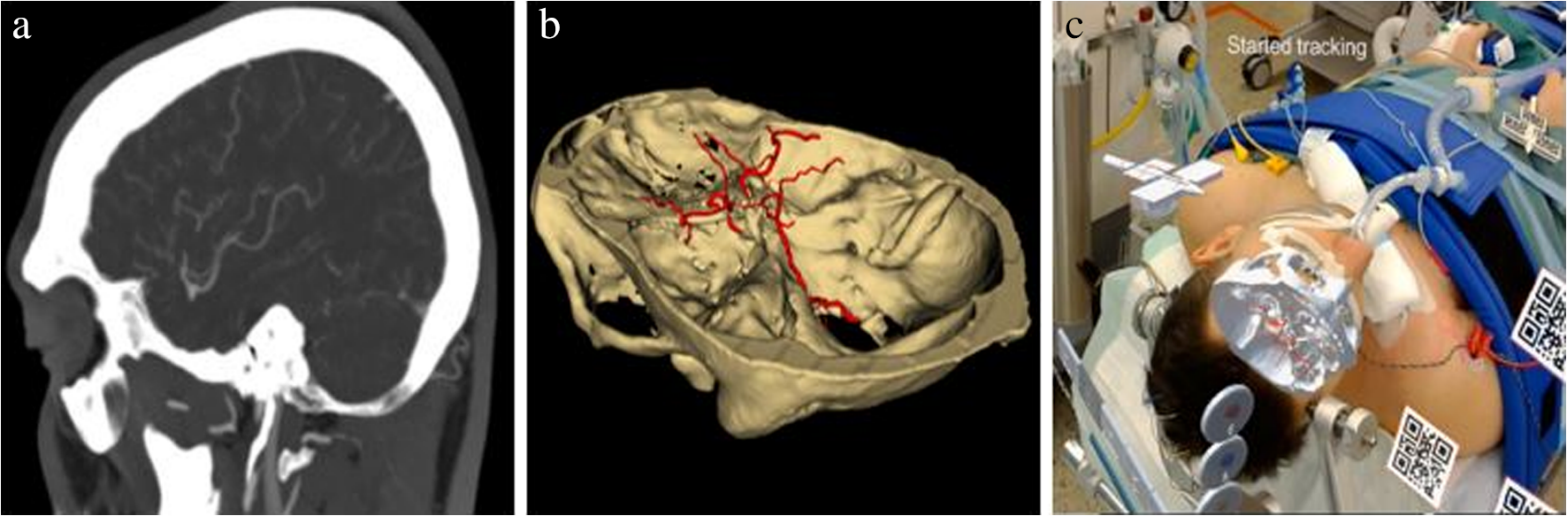
(a) CT-image of MCA aneurysm, sagittal view (b) Holograms of skull base and circle of Willis including MCA aneurysm on the left side (c) Holograms after registration as seen through the HoloLens

Patient 3 was a 40-year-old male who underwent a clipping of an MCA aneurysm on the left side. Based on CT-imaging (Fig. 6a), holograms were made of the skin, skull, skull base, and circle of Willis (Fig. 6b). Seven anatomical landmarks were chosen for registration: medial and lateral canthus of both eyes, nasal bridge, proximal part of the philtrum, and a distinctive part of the antihelix on the left side. Two registrations were performed with both an FRE of 7.0 mm (Fig. 6c). After the second registration, the bed was moved upwards. The reference array was tracked again, and the hologram repositioned with no change in accuracy of registration.

## Discussion

In this study, we presented three neurosurgical cases where we showed a proof-of-concept of our custom AR neuronavigation system. With this system, initial registration can be corrected for bed movements using a reference array. We measured the accuracy of initial point-based registration using FRE, and we evaluated the accuracy of registration correction after bed movement. We measured a mean FRE for initial registration of 8.55 mm. Patient tracking was successfully conducted in all cases.

Several indexes can be used to evaluate navigation accuracy. The target registration error (TRE) is considered of most interest to the surgeon and is often used to evaluate the accuracy of IR neuronavigation systems. When using a point-based registration method, which is most frequently used, FRE can be calculated automatically after registration. Although a specific FRE is not correlated with a specific TRE and therefore is not advised as an index to illustrate the accuracy of a specific registration [1], as an evaluation for the accuracy of a new neuronavigation system, it is a good indicator for navigation accuracy. Furthermore, FRE was used since there is still not a golden standard for measuring the TRE intraoperatively in a holographic scene. This is also why we did not collect quantitative data on the accuracy of the reference array. We acknowledge that determining the accuracy of the registration correction using visual observation is subjected to observation bias. However, effort is put into creating such a method so in the future we can conduct measurements in a lab environment and collect quantitative data. Furthermore, to minimize disruption of the OR workflow, we conducted only 2 registrations per patient with 2 subsequent FRE measurements. Considering the current variability between different registrations on the same patient, a larger sample of registrations per patient would be preferred for future experiments.

Although the technical workflow functioned as desired, the accuracy of initial registration was not adherent to the standard necessary for the clinical use of a neuronavigation system. Moreover, we recognized several technical limitations when using the software.

CN uses retroreflective spheres that can be tracked by an IR camera. In standard clinical practice, initial registration is conducted using an IR tracked probe on either anatomical landmarks, fiducial stickers, or surface matching. The accuracy of registration in IR-based neuronavigation is highly dependent on the method of registration: anatomical landmarks and surface matching are not as accurate as registration with fiducial adhesives [8, 9, 11, 14, 15]. For all methods of registration, a reference array with retroreflective spheres fixated to the head clamp or patient’s head is used to correct registration after intraoperative bed movement.

The concept of patient tracking for image-space is now implemented in our previously designed holographic neuronavigation system with the use of a 3D printed reference array with visual markers. To the best of our knowledge, we are the first presenting such a system. The system has several advantages when compared to CN. First, a stereoscopic 3-D hologram is directly superimposed on the patient, allowing a surgeon to directly assess the internal anatomy in relation to the real patient. Second, it provides a direct superimposition of the hologram in the working field during the macroscopic part of the procedure which in CN can only be achieved while using the microscope which is not preferred during that stage. This has ergonomic advantages when compared to a separate side-screen, improving the efficiency of the workflow and diminishing attention shifts.

Several problems that we described when using the previous version of our neuronavigation software still persisted. Despite improvements on probe design, initial registration was relatively inaccurate. We expect the usage of anatomical landmarks for registration to be a major contributor to this problem. Anatomical landmarks were used since this is the clinical standard in our department for registration when using an IR neuronavigation system, and as a proof-of-concept for the software, extra imaging for using adhesive fiducials was deemed too much of a burden for the patients. Anatomical landmarks account for a relatively large fiducial localization error which is the accuracy in which the fiducial points can be localized. Therefore, the point-clouds will not match as accurately as compared when using fiducial stickers or bone screws where the fiducials can be more accurately targeted. Moreover, probe tracking was inaccurate when registering anatomical points under suboptimal lighting or behind the head clamp. The latter we solved for the third patient by choosing more optimally placed anatomical landmarks. This way, the anatomical landmarks were easier to reach and the navigation accuracy improved. To improve registration further, in the future, we aim to use adhesive fiducials when possible. Furthermore, we are exploring methods similar to mask registration where the patient’s head can move freely during navigation. For this, a single marker will be adhered to the patient’s head which then will be used as reference point for the registration. This will allow constant tracking and correction of the holographic scene.

Problems with hologram stability were also still present. When large objects moved within the field-of-view of the AR-HMD before the hologram was matched on the reference array, the device could lose its spatial orientation. This could lead to severe drifting of the hologram. We plan to improve virtual “anchoring” of the hologram to recognizable geometrical static shapes in the real environment. Additionally, we expect future AR-HMDs to improve spatial mapping, which could lead to better hologram stability.

During the procedure, some functional difficulties regarding the usage of the device were reported. It was often difficult to find an optimal screen brightness and hologram opacity to allow the user to see both the hologram and patient clearly. This may cause difficulties for the surgeon when wearing the AR-HMD while marking the incision or performing a technical maneuver. This issue may be resolved by using more translucent hologram shaders or by moving the hologram to a floating position above the work field when performing a complex action.

## Conclusion

This early report shows a proof of principle of intraoperative patient tracking using a standalone holographic neuronavigation system. The navigation accuracy, hologram stability, and functional difficulties should be further optimized for the system to be clinically applicable. However, it is very likely that this 3-D technology will be incorporated in future neurosurgical workflows because of the distinct advantages it provides for neurosurgeons.

## Supplementary information

Online resource 1 Video showing the complete workflow in all three patients of the registration process and patient tracking after bed movement. Important notes: 1) When using the AR-HMD, there is a large offset between the user’s eyes and the camera used for video capture. This causes major visual registration inaccuracies not present in the real system. 2) As this video is captured and displayed monoscopically, the hologram seems to float over the real patient. When wearing the AR-HMD, a stereoscopic display of holograms creates an illusion of depth, which increases the quality of visualization. (MP4 309,339 kb)

## Abbreviations

AR: augmented reality CN conventional neuronavigation FRE fiducial registration error HMD head-mounted display IR infrared TRE target registration error
